# Promoter methylation of Wnt-antagonists in polypoid and nonpolypoid colorectal adenomas

**DOI:** 10.1186/1471-2407-13-603

**Published:** 2013-12-19

**Authors:** Quirinus JM Voorham, Jerry Janssen, Marianne Tijssen, Suzanne Snellenberg, Sandra Mongera, Nicole CT van Grieken, Heike Grabsch, Martin Kliment, Bjorn J Rembacken, Chris JJ Mulder, Manon van Engeland, Gerrit A Meijer, Renske DM Steenbergen, Beatriz Carvalho

**Affiliations:** 1 Department of Pathology, VU University Medical Center, PO Box 7057, 1007 MB Amsterdam, The Netherlands; 2 Department of Pathology, University Maastricht, Maastricht, The Netherlands; 3 GROW, School for Oncology and Developmental Biology, Maastricht University Medical Center, Maastricht, The Netherlands; 4 Pathology and Tumour Biology, Leeds Institute of Molecular Medicine, University of Leeds, Leeds, UK; 5 Gastroenterology, Hospital Vitkovice, Ostrava, Czech Republic; 6 Centre for Digestive Diseases, Leeds General Infirmary, Leeds, UK; 7 Departmentt of Gastro-enterology, VU University Medical Center, Amsterdam, The Netherlands

**Keywords:** qMSP, Colon, Superficial elevated, Flat adenoma, CpG-island, Epigenetic, Hypermethylation, Wnt-signaling pathway, APC

## Abstract

**Background:**

Nonpolypoid adenomas are a subgroup of colorectal adenomas that have been associated with a more aggressive clinical behaviour compared to their polypoid counterparts. A substantial proportion of nonpolypoid and polypoid adenomas lack *APC* mutations, *APC* methylation or chromosomal loss of the *APC* locus on chromosome 5q, suggesting the involvement of other Wnt-pathway genes. The present study investigated promoter methylation of several Wnt-pathway antagonists in both nonpolypoid and polypoid adenomas.

**Methods:**

Quantitative methylation-specific PCR (qMSP) was used to evaluate methylation of four Wnt-antagonists, *SFRP2*, *WIF-1*, *DKK3* and *SOX17* in 18 normal colorectal mucosa samples, 9 colorectal cancer cell lines, 18 carcinomas, 44 nonpolypoid and 44 polypoid adenomas. Results were integrated with previously obtained data on *APC* mutation, methylation and chromosome 5q status from the same samples.

**Results:**

Increased methylation of all genes was found in the majority of cell lines, adenomas and carcinomas compared to normal controls. *WIF-1* and *DKK3* showed a significantly lower level of methylation in nonpolypoid compared to polypoid adenomas (p < 0.01). Combining both adenoma types, a positive trend between *APC* mutation and both *WIF-1* and *DKK3* methylation was observed (p < 0.05).

**Conclusions:**

Methylation of Wnt-pathway antagonists represents an additional mechanism of constitutive Wnt-pathway activation in colorectal adenomas. Current results further substantiate the existence of partially alternative Wnt-pathway disruption mechanisms in nonpolypoid compared to polypoid adenomas, in line with previous observations.

## Background

Colorectal cancer (CRC) results from the accumulation of multiple alterations in the (epi) genome of the epithelial cells that line the large intestine. These events first give rise to an adenoma that, in a minority of cases progresses into an invasive and potentially metastasizing adenocarcinoma.

The terms polyp and adenoma have long been used as synonyms. However, more recently it was recognized that other phenotypes exist besides the traditional polypoid colorectal adenomas. Already in 1985 Muto *et al.* described a lesion in the large intestine that was termed ‘small flat adenoma’ [1]. These nonpolypoid adenomas were, until quite recently, considered rare in Western countries. In Japan, on the other hand, they have been reported to represent up to 40% of all colorectal adenomas or early carcinomas [2,3]. Current studies in Western countries, using advanced endoscopic imaging techniques, have reported similar incidences of nonpolypoid lesions as in the East [4-7]. Nonpolypoid lesions have been associated with a more aggressive behavior, are considered more likely to contain advanced histology [7,8] and are expected to have a different tumor biology [3,9].

Well known events during the progression of adenoma to carcinoma are the loss of tumor suppressor *TP53*, and constitutive activation of *KRAS* and the Wnt-pathway [10]. Wnt-pathway activation represents a critical early event in colorectal tumorigenesis and primarily results from inactivating mutations in its gatekeeper *APC*[11,12]. Recently, we found that nonpolypoid adenomas display less *APC* mutations and simultaneously more frequent chromosome 5q loss (locus of *APC*) compared to polypoid adenomas [13,14]. *APC* silencing by promoter hypermethylation occurred at similar frequencies in both phenotypes (Voorham *et al.*, submitted). However, in a substantial part of adenomas of both phenotypes no direct *APC* disruption was observed. Next to activation of the Wnt-signalling pathway via inactivation of the *APC* gene (e.g. by mutation, deletion or hypermethylation), methylation-mediated silencing of other upstream Wnt-signal regulating genes may present an alternative mechanism of constitutive Wnt-pathway activation in CRC [15,16]. Methylation plays an important role in CRC development and many genes have altered methylation patterns in the tumor compared to normal colon mucosa.

We aimed to investigate the contribution of methylation of a number of Wnt-regulators other than *APC* in both nonpolypoid and polypoid adenomas. To this end, four genes were selected known to have an antagonistic effect on the Wnt-pathway, which have been described before to be frequently methylated in CRC; Secreted Frizzled-Related Protein-2 (*SFRP2*), Wnt Inhibitory Factor-1 (*WIF-1*), Dickkopf-3 (*DKK3*) and SRY-Box-17 (*SOX17*) [17-24]. Promoter methylation of these four genes was determined using quantitative methylation-specific PCR (qMSP) [25] in a well-characterized series of both nonpolypoid and polypoid adenomas, and findings were related to previously obtained data on *APC* mutation, *APC* promoter methylation and genomic loss of the *APC* locus in the same adenomas.

## Methods

### Cell cultures

A panel of 9 CRC cell lines (Caco2, Colo205, Colo320, HCT116, HT29, SW480, SW620, LS174T and LS513) was used in this study. Colo205, Colo320, HCT116, HT29, SW480, SW620, LS174T and LS513 were cultured in DMEM (Lonza Biowhittaker, Verviers, Belgium) supplemented with 10% fetal calf serum (FCS) (Hyclone, Perbio, Etten-Leur, The Netherlands). Caco2 was cultured in RPMI1640 (Lonza Biowhittaker) supplemented with 20% FCS. Both cell culture media were supplemented with 2 mM L-Glutamine, 100 IU/ml sodium-penicillin (Astellas Pharma B.V., Leiderdorp, The Netherlands) and 100 μg/ml streptomycin (Fisiopharma, Palomonta (SA), Italy). The cervical cancer cell line CaSki was used as positive control and cultured as described before [26]. All cell lines were cultured using coated flasks and dishes (Greiner Bio-One, Frickenhausen, Germany).

### Ethical statement

Collection, storage and use of archival tissue and patient data were performed in compliance with the “Code for Proper Secondary Use of Human Tissue in the Netherlands” (http://www.fmwv.nl and http://www.federa.org). This study was approved by the VU University medical center (2011-03), the Leeds University (CA02/014 Leeds (West)) and the Hospital Vitkovice (EK/140/10).

This study followed the ethical guidelines of the Institutional Review Board (IRB). The IRB waived the need for consent for use of the archive samples, and the samples were analyzed anonymously.

### Patient and sample selection

Formaldehyde-fixed, paraffin-embedded (FFPE) colorectal tissue samples were collected at three different institutes; Leeds General Infirmary in Leeds, UK, Hospital Vitkovice in Ostrava, Czech Republic and VU University medical center in Amsterdam, The Netherlands [5,13,27]. Patients with a hereditary form of CRC, inflammatory bowel disease were excluded. The final series contained 44 nonpolypoid adenomas, 44 polypoid adenomas and 18 carcinomas. Normal colorectal mucosa was collected from age matched non-cancerous patients. Classification of the adenomas was performed using the Paris classification [28]. A summary of all clinical characteristics is listed in Table 1.

**Table 1:** Clinical characteristics of the patients used in this study

	**Normal colorectal mucosa (n = 18)**	**Nonpolypoid adenomas (n = 44)**	**Polypoid adenomas (n = 44)**	**Carcinomas (n = 18)**
Gender M/F	6/12	24/20	22/22	8/10
Age (years)	71.33 (49-84)	70.77 (50-87)	70.98 (40-90)	71.50 (51-91)
Location				
Left colon		14	40	7
Right colon		30	4	10
Size		17.30 (5-100)	19.39 (6-64)	N/A
Paris classification				
0-IIa		18		
LST-G		7		
LST-NG		19		
0-Ip			44	
Histology/AC-stage				
Tubular/B1		21	22	2
Tubulovillous/B2		20	18	9
Villous/B3		2	4	1
Serrated/C2		1		5
C3				1
Dysplasia				
Mild (LGD)		2	1	
Moderate (LGD)		38	36	
Severe (HGD)		4	7	
Chromosome 5q loss		8/38	1/30	
APC methylation		34/41	29/44	
APC mutation		16/44	26/44	

Right colon includes caecum, ascending and transverse colon; Left colon includes sigmoid, descending and splenic flexure; 0-IIa, slightly elevated adenoma; LST-G, Granular lateral spreading type adenoma; LST-NG, Non-granular lateral spreading type adenoma; 0-Ip, polypoid adenoma; AC-stage, Astler-Coller stage; LGD, low grade dysplasia; HGD, high grade dysplasia.

### DNA and RNA isolation

DNA and RNA from cell lines was isolated using TRIzol Reagent (Life Technologies, Breda, The Netherlands) according to the manufacturers’ instructions [29]. DNA from FFPE material was isolated after macro-dissection as described before [14].

### Quantitative methylation specific PCR (qMSP)

DNA methylation analysis of *SFRP2*, *WIF-1*, *DKK3* and *SOX17* was performed using quantitative methylation specific PCR (qMSP) as described before [25,30]. All samples were tested in duplicate and average Ct values were used for further analysis. Samples with delta Ct values between duplicates more than 1.5 were excluded.

In addition, the modified, unmethylated sequence of the housekeeping gene B-actin (ACTB) was amplified as a reference to verify sufficient DNA quality and successful DNA modification [31]. Samples with Ct-values > 32 for ACTB were excluded from further analysis. In all qMSP runs, a negative (non-bilsulfite-treated cell line DNA (CaSki)) and a positive (bisulfite-treated CaSki DNA) control were included. For all samples the delta Ct ratio between the gene of interest and ACTB was calculated using the 2^-ΔCt^ method [32]. The upper limit of the 99% confidence interval of normal controls was used as cut-off value to determine methylation positivity. The reproducibility of these assays has been demonstrated previously [25].

### Relation between methylation and gene expression

CaSki cells were incubated with 0.2 and 5 μM 5-aza-2′-deoxycytidine (DAC; Sigma Chemical Co, St Louis, MO, USA) diluted in PBS for five days. All incubations were performed in duplicate, and cells were directly harvested for DNA and RNA isolation. Gene expression was evaluated by RT-PCR as previously described [30].

### Statistical analysis

Statistical analysis was performed using SPSS 20. We used a significance level of p < 0.013 (0.05/4 genes), to adjust for multiple testing according to the correction suggested by Bonferroni [33]. Comparisons between the methylation levels in CRC cell lines and normal colon were done using a non-parametric Mann-Whitney U test. After that, a positivity score was performed using a cut off level based on the upper limit of the 99% confidence interval of the normal controls. All group comparisons were performed using two-sided chi-square or Fisher exact test when expected values in the cross table are below 10. In the overview tables it is indicated which test was applied.

Logistic regression was used to examine the relationship between *WIF-1* methylation and the independent variables phenotype, location, *APC* methylation, 5q loss and *APC* mutation. First the univariate relationships between gene methylation and the dependent variables were examined. Multivariate analyses were performed including all variables with a univariate p-value of less than 0.1. Next, we used a stepwise procedure and removed the variable with the largest p-value in each step, until only variables with a p-value < 0.05 remained in the multivariate model.

## Results

### Promoter methylation in normal colon and CRC cell lines

**Figure 1:**
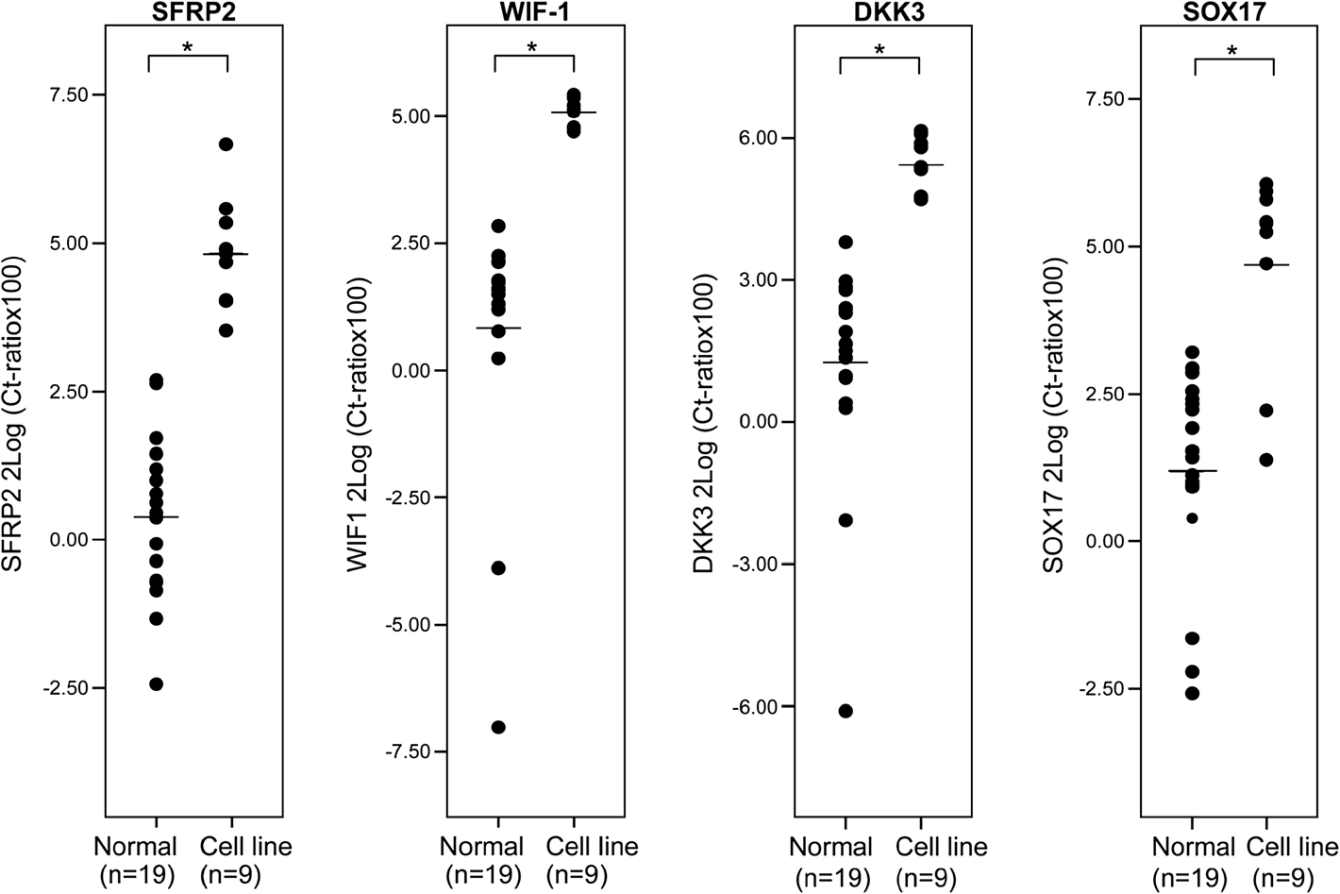
**Promoter methylation status in normal colon mucosa and in colorectal cancer cell lines.** Scatter plots of the levels of *SFRP2*, *WIF-1*, *DKK3*, *SOX17* methylation. On the y-axes levels of DNA methylation are shown. The median is indicated with a black line. Asterisks indicate a statistical significant difference.

Comparison of the *SFRP2*, *WIF-1*, *DKK3* and *SOX17* promoter methylation levels between nine CRC cell lines and eighteen normal colon mucosa samples revealed significantly elevated methylation levels in CRC cells for all four genes (p = 0.00001, p = 3.1*10^-5^, p = 3.1*10^-5^ and p = 0.001 for *SFRP2*, *WIF-1*, *DKK3* and *SOX17*, respectively). For *SFRP2, WIF-1* and *DKK3* increased methylation levels were observed in all nine CRC cell lines, whereas *SOX17* showed higher methylation levels in all but two cell lines (LS513 and LS174T) (Figure 1).

Methylation lead to decreased expression, as upon treatment with demethylating agents, an increase in expression was observed for *SFRP2*, *DKK3* and *SOX17* but not for *WIF-1* (Additional file 1: Figure S1).

### Promoter methylation in carcinomas, polypoid and nonpolypoid adenomas

Since the findings in cell lines are supportive of a role of promotor methylation of these genes in colorectal carcinogenesis, we next investigated a series of tissue specimens consisting of 18 carcinomas, 44 nonpolypoid and 44 polypoid adenomas.

**Figure 2:**
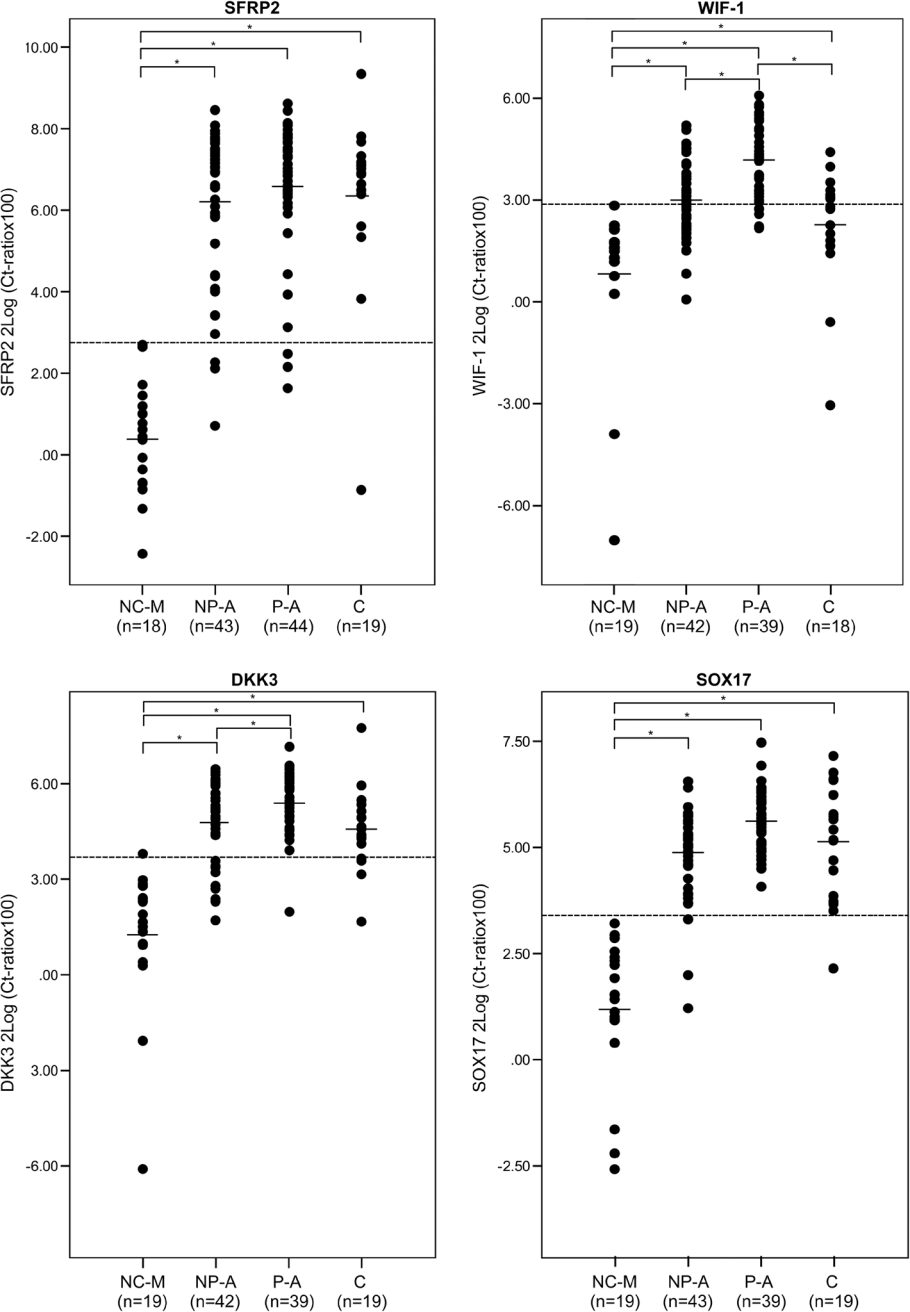
**Promoter methylation status in normal colon mucosa and different lesion types.** Scatter plots of the levels of *SFRP2*, *WIF-1*, *DKK3*, *SOX17* methylation. On the y-axis levels of DNA methylation are shown; On the x-axis normal colon mucosa (NC-M), nonpolypoid adenomas (NP-A), polypoid adenomas (P-A) and carcinomas (C) are indicated. The dotted line indicates the methylation cut off value based on the 99% CI of normal colorectal mucosa. Asterisks indicate a statistical significant difference.

Increased methylation levels for all four genes were detectable in all carcinomas and in both polypoid and nonpolypoid adenomas (Figure 2).

Interestingly, methylation levels in nonpolypoid adenomas were more similar to those observed in carcinomas than those in polypoid adenomas. No relation in methylation levels of any of the four genes and the different carcinoma stages was observed (data not shown).

**Figure 3:**
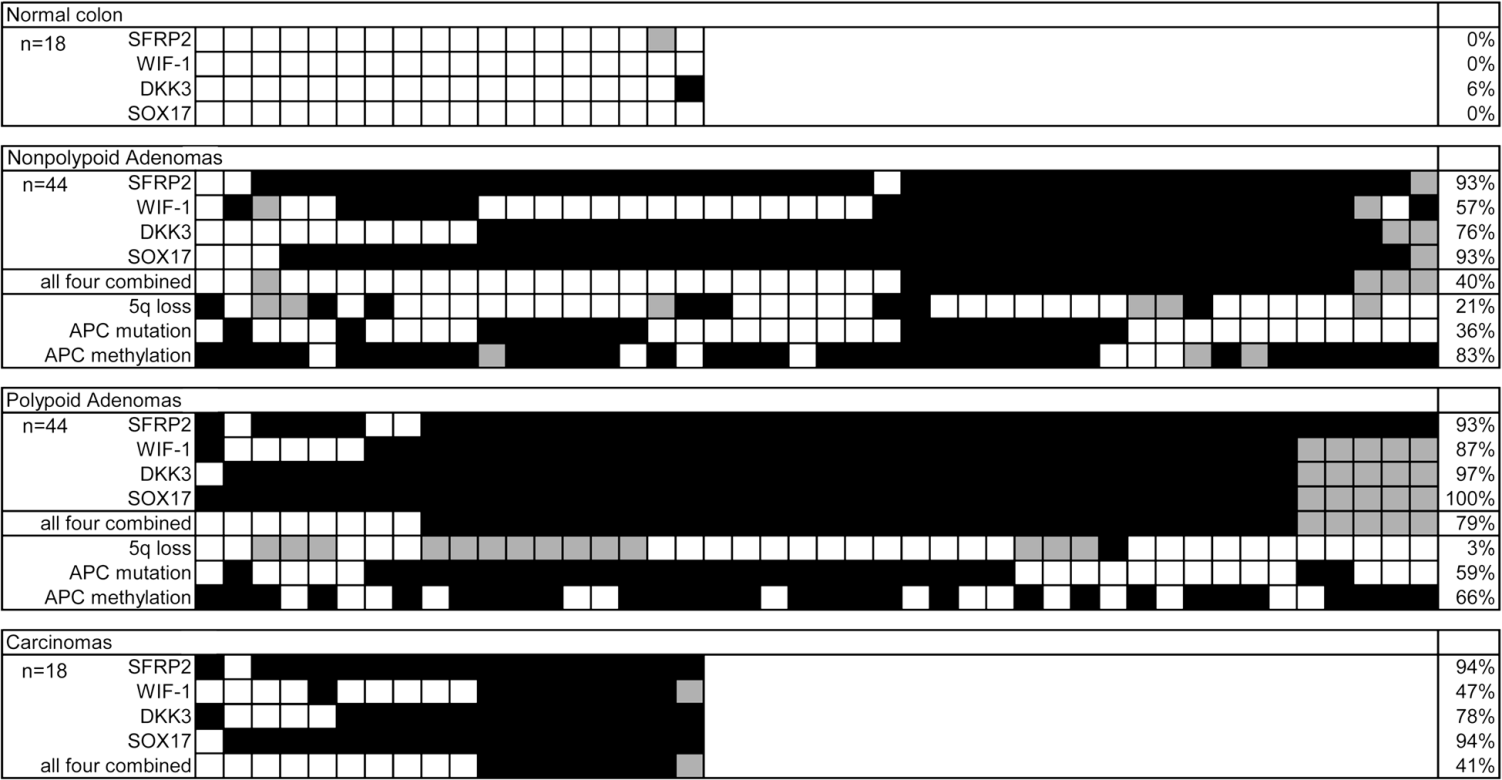
**Overview of DNA promoter methylation results of *SFRP2*, *WIF-1*, *DKK3 *and *SOX17*.** Methylation results of *SFRP2*, *WIF-1*, *DKK3* and *SOX17* are shown in relation to previous results on chromosome 5q loss [14], *APC* mutation [13] and *APC* methylation (Voorham *et al.* submitted) in nonpolypoid and polypoid adenomas. Black box; event (methylation, 5q loss, *APC* mutation, White box; no event (no methylation, no chromosome 5q loss, *APC* wild type), Grey box; no results.

To dichotomize the qMSP results into positive or negative for methylation, a cut off was calculated for each gene based on the 99% confidence interval of the normal controls (Figure 3). Significantly increased positivity rates were observed for all four genes in both types of adenomas and in carcinomas compared to normal colorectal mucosa (p < 0.003, Additional file 2: Table S1). Interestingly, *DKK3* and *WIF-1* methylation frequencies were significantly higher in polypoid adenomas (*DKK3*; 97% (38/39), *WIF-1*; 87% (34/39)) compared to nonpolypoid adenomas (*DKK3*; 76% (32/42), *WIF-1*; 57% (24/42); p = 0.005 and p = 0.003, respectively). *WIF-1* methylation was also significantly higher in polypoid adenomas compared to carcinomas (*WIF-1*; 47% (8/17), p = 0.003).

When the methylation results of the four Wnt-antagonists were combined into one value that was positive if all four markers were methylated 79% (31/39) of the polypoid adenomas were positive in comparison to only 40% (16/40) of the nonpolypoid adenomas (p = 0.0004), indicating a lower level of Wnt-antagonist methylation in nonpolypoid adenomas in general (Figure 3, Additional file 2: Table S1).

### Promoter methylation in relation to anatomical location

To investigate the relation between methylation of *SFRP2*, *WIF-1*, *DKK3* and *SOX17* and the anatomical location of the adenoma, we divided all the adenomas into left- and right-sided adenomas. This showed no statistical difference for the investigated genes, except for *WIF-1* methylation, which showed more methylation in the left colon 82% (40/49) compared to the right colon 56% (18/32), p = 0.01 (Table 2).

**Table 2:** **Promoter methylation frequencies of *SFRP2*, *WIF-1*, *DKK3 *** **and *SOX17 *** **in relation to location**

	**Left nonpolypoid adenomas**	**Right nonpolypoid adenomas**	**p-value**	**Left polypoid adenomas**	**Right polypoid adenomas**	**p-value**	**All adenomas left**	**All adenomas right**	**p-value**
*SFRP2*	79% (11/14)	100% (29/29)	0.3**	93% (37/40)	100% (4/4)	1**	89% (48/54)	100% (33/33)	0.08**
*WIF-1*	69% (9/13)	52% (15/29)	0.3**	86% (31/36)	100% (3/3)	1**	82% (40/49)	56% (18/32)	0.013*
*DKK3*	64% (9/14)	82% (23/28)	0.3**	97% (35/36)	100% (3/3)	1**	88% (44/50)	84% (26/31)	0.7**
*SOX17*	86% (12/14)	97% (28/29)	0.2**	100% (36/36)	100% (3/3)	-	96% (48/50)	97% (31/32)	1**
All four combined	38% (5/13)	41% (11/27)	0.9*	78% (28/36)	100% (3/3)	1**	67% (33/49)	47% (14/30)	0.07**

Results are shown for polypoid adenomas, nonpolypoid adenomas and all adenomas together, divided into samples that were distal (left) or proximal (right).

All four combined; is considered positive if all four genes showed methylation. *chi-square based p-value **Fisher exact based p-value.

### Promoter methylation combined with other molecular events

Methylation of the Wnt-antagonists may provide an alternative mechanism of Wnt-pathway activation next to *APC* mutations, methylation and loss of the *APC* locus on chromosome 5q. Therefore, we combined the promoter methylation results for *SFRP2*, *WIF-1*, *DKK3* and *SOX17*, in polypoid and nonpolypoid adenomas, with previously obtained molecular data on *APC* mutation [13], *APC* methylation (Voorham *et al*., submitted) and chromosome 5q loss [14] in the same samples. For *APC* methylation as well as for chromosome 5q loss, no relation was found with the promoter methylation results for *SFRP2*, *WIF-1*, *DKK3* and *SOX17* when combining both adenomas types or in nonpolypoid adenomas or polypoid adenomas, separately (Tables 3 and 4). For *APC* mutation, a positive trend with *WIF-1* as well as with *DKK3* methylation was observed (Table 5). Of the *APC* mutated adenomas 83% (33/40) showed *WIF-1* methylation and of the *APC* wild type adenomas 61% (25/41) showed *WIF-1* methylation (p = 0.048). For *DKK3*, 95% (38/40) of the *APC* mutated samples showed *DKK3* methylation whereas only 78% (32/41) showed *DKK3* methylation in the *APC* wild type adenomas (p = 0.026) (Table 5). When we combined *APC* methylation, *APC* mutation and chromosome 5q loss together into one value called *APC* disrupting event, no significant difference was found (Additional file 2: Table S2).

**Table 3:** **Promoter methylation frequencies of *SFRP2*, *WIF-1*, *DKK3 *** **and *SOX17 *** **in relation with *APC *** **methylation**

	**Nonpolypoid APC methylated**	**Nonpolypoid APC unmethylated**	**p-value**	**Polypoid APC methylated**	**Polypoid APC unmethylated**	**p-value**	**All adenomas APC methylated**	**All adenomas APC unmethylated**	**p-value**
*SFRP2*	91% (30/33)	100% (7/7)	1**	93% (27/29)	93% (14/15)	1**	92% (57/62)	96% (21/22)	1**
*WIF-1*	59% (19/32)	43% (3/7)	0.7**	93% (22/25)	93% (12/14)	1**	71% (41/57)	68% (15/21)	1*
*DKK3*	72% (23/32)	86% (6/7)	0.7**	96% (24/25)	100% (14/14)	1**	83% (47/57)	96% (20/21)	0.3**
*SOX17*	91% (30/33)	100% (7/7)	1**	100% (25/25)	100% (14/14)	-	95% (55/58)	100% (21/21)	0.6**
All four combined	37% (11/30)	43% (3/7)	1**	80% (20/25)	79% (11/14)	1**	56% (31/55)	67% (14/21)	0.4*

Results are shown for polypoid adenomas, nonpolypoid adenomas and all adenomas together, divided into samples that were *APC* methylated or *APC* unmethylated.

All four combined; is considered positive if all four genes showed methylation. *chi-square based p-value **Fisher exact based p-value.

**Table 4:** **Promoter methylation frequencies of *SFRP2*, *WIF-1*, *DKK3 *** **and *SOX17 *** **in relation with chromosome 5q loss**

	**Nonpolypoid 5q loss**	**Nonpolypoid no 5q loss**	**p-value**	**Polypoid 5q loss**	**Polypoid no 5q loss**	**p-value**	**All adenomas 5q loss**	**All adenomas no 5q loss**	**p-value**
*SFRP2*	75% (6/8)	97% (28/29)	0.1**	100% (1/1)	90% (26/29)	1**	78% (7/9)	93% (54/58)	0.2**
*WIF-1*	50% (4/8)	60% (18/30)	0.7**	100% (1/1)	92% (22/24)	1**	56% (5/9)	74% (40/54)	0.3**
*DKK3*	63% (5/8)	82% (23/28)	0.3**	100% (1/1)	96% (23/24)	1**	67% (6/9)	89% (46/52)	0.1**
*SOX17*	88% (7/8)	97% (28/29)	0.4**	100% (1/1)	100% (24/24)	-	89% (8/9)	98% (52/53)	0.3**
All four combined	25% (2/8)	43% (12/28)	0.4**	100% (1/1)	66% (19/24)	1**	33% (3/9)	60% (31/52)	0.2**

Results are shown for polypoid adenomas, nonpolypoid adenomas and all adenomas together, divided into samples that had a chromosome 5q loss (locus of *APC*) or samples that had no chromosome 5q loss.

All four combined; is considered positive if all four genes showed methylation. **Fisher exact based p-value.

**Table 5:** **Promoter methylation frequencies of *SFRP2*, *WIF-1*, *DKK3 *** **and *SOX17 *** **in relation with *APC *** **mutation**

	**Nonpolypoid APC mutated**	**Nonpolypoid APC WT**	**p-value**	**Polypoid APC mutated**	**Polypoid APC WT**	**p-value**	**All adenomas APC mutated**	**All adenomas APC WT**	**p-value**
*SFRP2*	94% (15/16)	93% (25/27)	1**	89% (23/26)	100% (18/18)	0.3**	91% (38/42)	96% (43/45)	0.4**
*WIF-1*	63% (10/16)	54% (14/26)	0.6**	96% (23/24)	73% (11/15)	0.06**	83% (33/40)	61% (25/41)	0.03*
*DKK3*	88% (14/16)	69% (18/26)	0.3**	100% (24/24)	93% (14/15)	0.4**	95% (38/40)	78% (32/41)	0.03*
*SOX17*	94% (15/16)	93% (25/27)	1**	100% (24/24)	100% (15/15)	-	98% (39/40)	95% (40/42)	1**
All four combined	50% (8/16)	33% (8/24)	0.3*	88% (21/24)	67% (10/15)	0.2**	73% (29/40)	46% (18/39)	0.017*

Results are shown for polypoid adenomas, nonpolypoid adenomas and all adenomas together, divided into samples that were *APC* mutated or *APC* wild type.

All four combined; is considered positive if all four genes showed methylation. *chi-square based p-value **Fisher exact based p-value.

### Multivariate analyses

To investigate the possible interaction between the different variables (phenotype, location, *APC* mutation, *APC* methylation and chromosome 5q loss), a multivatiate analysis was performed for *WIF-1* methylation. For the genes *SFRP2*, *DKK3* and *SOX17*, we were unable to perform a valid multivariate analysis, due to the limited number of unmethylated samples[34].

For the *WIF-1* gene, we first performed univariate analyses showing that phenotype (p-value = 0.004), location (p-value = 0.016) and *APC* mutation (p = 0.035) were related to *WIF-1* methylation. In the multivariate analysis, location and *APC* mutation were removed from the model (due to high p-values), leaving only phenotype in the model. This suggests that phenotype is the major contributor to the observed difference in *WIF-1* methylation in our samples.

## Discussion

The present study focussed on promoter methylation of four known Wnt-pathway antagonists (*SFRP2*, *WIF-1*, *DKK3* and *SOX17*), in polypoid and nonpolypoid adenomas, and its possible association with other molecular events that can play a role in Wnt-pathway activation. All four Wnt-antagonists showed significant increased methylation in CRC cell lines, carcinomas as well as in nonpolypoid and polypoid adenomas compared to normal colon mucosa. A functional relation between methylation and gene silencing was shown for *SFRP2*, *DKK3* and *SOX17*.

To the best of our knowledge methylation of *SFRP2, DKK3* and *SOX17* has not been described in nonpolypoid adenomas before. Consistent with our findings, *WIF-1* was described to be less frequent methylated in nonpolypoid lesions compared to polypoid ones [9,35].

The higher methylation of all four Wnt-antagonists in CRC cell lines as well as carcinomas, compared to normal colon mucosa, confirms current literature [17,19-23].

Interestingly, we found lower *WIF-1* methylation frequencies in carcinomas compared to polypoid adenomas (*WIF-1*; 47% versus 87%) but not compared to nonpolypoid adenomas. Lower levels of methylation in carcinomas compared to adenomas have been described before for *WIF-1*[17] but also for other genes, such as p14 [36] and *ESR1*[17]. This may suggest that methylation of *WIF-1* is less important in carcinomas or that silencing of these genes in carcinomas is achieved by other changes to the DNA [15]. We did not find a relation between methylation and mRNA expression for *WIF-1*, indicating that *WIF-1* gene expression might be regulated by more complex regulatory mechanisms, potentially including histone modification.

For *DKK3* methylation a positive relation with higher CRC stages was described [37]. This could not be confirmed in our study, which may be explained by the limited number of carcinomas investigated. For *WIF-1* methylation no relation with CRC stage was observed by either Aguilera *et al.*[37] or us.

Analysis of the relation of methylation of all four genes with previously published results on *APC* disrupting events (*APC* mutation, *APC* methylation and chromosome 5q loss, including the locus of *APC*) revealed a positive trend between *WIF-1* and *DKK3* methylation and *APC* mutation. Although, the role of *WIF-1* and *DKK3* in the Wnt-signaling pathway is still poorly understood, these data may suggest that methylation of these Wnt-antagonists is complementing *APC* disruption and acts synergistically [11,38].

Left and right CRCs have been suggested to be different clinicopathological entities [39,40] Right CRCs occur at an older age, predominantly in women and are characterized by a high frequency of microsatellite instability and hypermethylation, whereas left CRCs occur predominantly in men and are characterized by chromosomal instability [40]. A recent study revealed a (partly) distinct methylation pattern in left- and right-sided adenomas [41]. However, for some genes methylation levels were higher in right-sided adenomas whereas for others methylation levels were higher in left-sided adenomas. In the current study we observed more frequent *WIF-1* methylation in left-sided adenomas compared to right-sided adenomas. All other three genes were location-independent.

Next to the above mentioned observation that *WIF-1* methylation was more frequent in adenomas from the left colon, *WIF-1* methylation was also higher in polypoid adenomas compared to nonpolypoid adenomas. This could introduce a bias in our analysis, since it is reported that nonpolypoid adenomas occur more frequently in the right colon compared to the left colon [42]. To further investigate this, we performed a multivariate analysis including phenotype and location but also *APC* mutation, *APC* methylation and chromosome 5q loss. From this analysis it became clear that phenotype was the main contributor to the observed difference between polypoid and nonpolypoid adenomas.

In the current study we had to restrict our analysis to a candidate gene approach, given the fact that the nonpolypoid adenomas studied are very small and concerned FFPE material, as of which only a few methylation events could be studied. A genome-wide methylation profiling approach may reveal further distinctions between both types of adenomas.

## Conclusion

Methylation of *SFRP2*, *WIF-1*, *DKK3* and *SOX17* was significantly higher in carcinomas as well as both types of adenomas compared to normal colorectal mucosa. We found higher levels of methylation for *WIF-1* and *DKK3* in polypoid adenomas compared to nonpolypoid adenomas. These results further substantiate differences in Wnt-pathway disruption as already observed previously for *APC* mutation rate and *APC* loss in nonpolypoid adenomas compared to polypoid adenomas.

## Abbreviations

APC: Adenomatous polyposis coli; CRC: Colorectal cancer; FFPE: Formaldehyde-fixed, paraffin-embedded; qMSP: Quantitive methylation specific PCR; MSP: Methylation specific PCR.

## Competing interests

The authors declare that they have no competing interests.

## Authors’ contributions

Study concept and design, BC, RS, CM, MvE, GM; acquisition of data, QV, JJ, MT, SM, SS; analysis and interpretation of data, QV, JJ, SS; drafting of the manuscript, QV, JJ; critical revision of the manuscript, BC, RS, GM, MvE, HG; statistical analysis, QV, JJ, SS; obtained funding; CM, MvE, GM; technical support, MT, SM, SS; material support, NvG, MK, HG, BR; study supervision, GM, BC, RS. All authors read and approved the final manuscript.

## Pre-publication history

The pre-publication history for this paper can be accessed here:

http://www.biomedcentral.com/1471-2407/13/603/prepub

## Supplementary Material

Additional file 1: Figure S1 *DKK3*, *SFRP2* and *SOX17* promoter methylation is associated with reduced expression. We evaluated whether *SFRP2*, *WIF-1*, *DKK3* and *SOX17* DNA methylation was inversely correlated with its gene expression. It was shown before that all four genes were methylated in CaSki cells [25] and therefore these cells were treated with the methylation inhibitor DAC. QMSP analysis revealed high levels of methylation of all four genes (panel A). Following DAC treatment a clear decrease in methylation was seen for *SFRP2*, *WIF-*1 and *SOX17* and to a somewhat lesser extent for *DKK3*. As shown in panel B, the decreased methylation after DAC treatment was correlated to an increase in *SFRP2*, *DKK3* and *SOX17* mRNA expression. The housekeeping gene SnRNP was used as control [30]. No effect on *WIF-1* mRNA expression was found after DAC treatment. Hence, methylation of *SFRP2*, *DKK3* and *SOX17* affects its gene expression. Click here for file

Additional file 2: Table S1 Promoter methylation frequencies of *SFRP2*, *WIF-1*, *DKK3* and *SOX17* in polypoid adenomas, nonpolypoid adenomas and carcinomas. **Table S2.** Promoter methylation frequencies of *SFRP2, WIF-1*, *DKK3* and *SOX17* in relation with an *APC* disrupting event. Results are shown for all adenomas, divided into samples that harbor an *APC* disrupting event or lack an *APC* disrupting event. Click here for file
