# Supplementary figures and images for: Promoter methylation of Wnt-antagonists in polypoid and nonpolypoid colorectal adenomas
Source: BMC Cancer. 2013 Dec 19;13:603. doi: 10.1186/1471-2407-13-603 (PMC3878219; doi:10.1186/1471-2407-13-603)

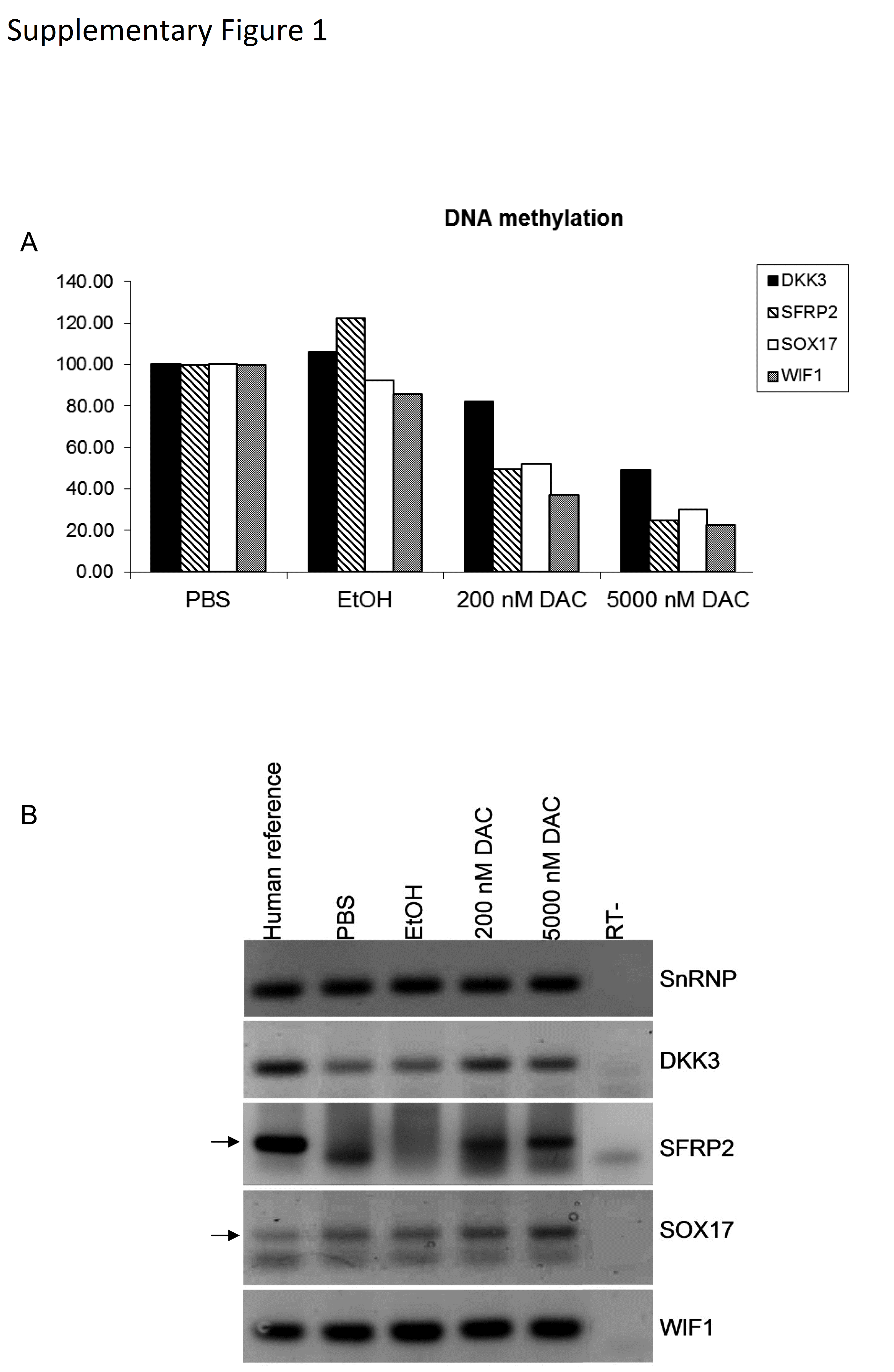

Supplement: Additional file 1: Figure S1 — DKK3, SFRP2 and SOX17 promoter methylation is associated with reduced expression. We evaluated whether SFRP2, WIF-1, DKK3 and SOX17 DNA methylation was inversely correlated with its gene expression. It was shown before that all four genes were methylated in CaSki cells [25] and therefore these cells were treated with the methylation inhibitor DAC. QMSP analysis revealed high levels of methylation of all four genes (panel A). Following DAC treatment a clear decrease in methylation was seen for SFRP2, WIF-1 and SOX17 and to a somewhat lesser extent for DKK3. As shown in panel B, the decreased methylation after DAC treatment was correlated to an increase in SFRP2, DKK3 and SOX17 mRNA expression. The housekeeping gene SnRNP was used as control [30]. No effect on WIF-1 mRNA expression was found after DAC treatment. Hence, methylation of SFRP2, DKK3 and SOX17 affects its gene expression. [file 1471-2407-13-603-S1.tiff]
