# Supplementary material for: Promoter methylation of Wnt-antagonists in polypoid and nonpolypoid colorectal adenomas
Source: BMC Cancer. 2013 Dec 19;13:603. doi: 10.1186/1471-2407-13-603 (PMC3878219; doi:10.1186/1471-2407-13-603)
Supplement: Additional file 2: Table S1 — Promoter methylation frequencies of SFRP2, WIF-1, DKK3 and SOX17 in polypoid adenomas, nonpolypoid adenomas and carcinomas. Table S2. Promoter methylation frequencies of SFRP2, WIF-1, DKK3 and SOX17 in relation with an APC disrupting event. Results are shown for all adenomas, divided into samples that harbor an APC disrupting event or lack an APC disrupting event. [file 1471-2407-13-603-S2.doc]

**SUPPLEMENTARY FILE**

**Supplementary Table 1; Promoter methylation frequencies of *SFRP2*, *WIF-1*, *DKK3* and *SOX17* in polypoid adenomas, nonpolypoid adenomas and carcinomas.**

|  | Normals | Nonpolypoid adenomas | Polypoid adenomas | Carcinomas | p-value (nonpolypoid vs normals) | p-value (polypoid vs normals) | p-value (carcinoma vs normals) | p-value (nonpolypoid vs polypoid) | p-value (polypoid vs carcinoma) | p-value (nonpolypoid vs carcinoma) |
| --- | --- | --- | --- | --- | --- | --- | --- | --- | --- | --- |
| *SFRP2* | 0% (0/17) | 93% (40/43) | 93% (41/44) | 94% (17/18) | 0* | 0* | 0* | 1** | 0.9** | 0.8** |
| *WIF-1* | 0% (0/18) | 57% (24/42) | 87% (34/39) | 47% (8/17) | 0* | 0* | 0.001** | 0.003* | 0.003** | 0.5* |
| *DKK3* | 6% (1/18) | 76% (32/42) | 97% (38/39) | 78% (14/18) | 0* | 0* | 0.00001* | 0.005* | 0.03** | 0.8** |
| *SOX17* | 0% (0/18) | 93% (40/43) | 100% (39/39) | 94% (17/18) | 0* | 0* | 0* | 0.24** | 0.3** | 0.8** |
| All four combined | 0% (0/17) | 40% (16/40) | 79% (31/39) | 41% (7/17) | 0.002** | 0* | 0.003** | 0.0004* | 0.005* | 0.9* |

All four combined; is considered positive if all four genes showed methylation. *chi-square based p-value **Fisher exact based p-value

**Supplementary Table 2; Promoter methylation frequencies of *SFRP2,* *WIF-1*, *DKK3* and *SOX17* in relation with an *APC* disrupting event.** Results are shown for all adenomas, divided into samples that harbor an *APC* disrupting event or lack an *APC* disrupting event.

|  | APC disrupting event positive | APC disrupting event negative | p-value |
| --- | --- | --- | --- |
| *SFRP2* | 100% (8/8) | 92% (70/76) | 1** |
| *WIF-1* | 63% (5/8) | 73% (51/70) | 0.7** |
| *DKK3* | 100% (8/8) | 84% (59/70) | 0.6** |
| *SOX17* | 100% (8/8) | 96% (68/71) | 1** |
| All four combined | 63% (5/8) | 59% (40/68) | 1** |

*APC* disrupting event is positive when a samples harbors an *APC* methylation, *APC* mutation or a chromosome 5q loss (locus of *APC*). All four combined; is considered positive if all four genes showed methylation. *chi-square based p-value **Fisher exact based p-value

**Supplementary Figure 1;** ***DKK3*, *SFRP2* and *SOX17* promoter methylation is associated with reduced expression.** We evaluated whether *SFRP2*, *WIF-1*, *DKK3* and *SOX17* DNA methylation was inversely correlated with its gene expression. It was shown before that all four genes were methylated in CaSki cells (van der Meide 2011) and therefore these cells were treated with the methylation inhibitor DAC. QMSP analysis revealed high levels of methylation of all four genes (panel A).  Following DAC treatment a clear decrease in methylation was seen for *SFRP2*, *WIF-*1 and *SOX17* and to a somewhat lesser extent for *DKK3*. As shown in panel B, the decreased methylation after DAC treatment was correlated to an increase in *SFRP2*, *DKK3* and *SOX17* mRNA expression. No effect on *WIF-1* mRNA expression was found after DAC treatment. Hence, methylation of *SFRP2*, *DKK3* and *SOX17* affects its gene expression.
